# Changes of Lignin Molecular Structures in a Modification of Kraft Lignin Using Acid Catalyst

**DOI:** 10.3390/ma9080657

**Published:** 2016-08-05

**Authors:** Sunghoon Kim, Seungtaek Oh, Jungmin Lee, Hyun-gyoo Roh, Jongshin Park

**Affiliations:** 1 Ore Co. Ltd., Seoul 04781, Korea; bv206driver@snu.ac.kr; 2 Hyosung, R&D Business Labs, Anyang 431-080, Korea; ost@hyosung.com; 3 Department of Biosystems & Biomaterials Science and Engineering, Research Institute of Agriculture and Life Sciences, Seoul National University, Seoul 151-742, Korea; archon04@snu.ac.kr (J.L.); ssangno@snu.ac.kr (H.R.)

**Keywords:** soft wood kraft lignin, lignin-carbohydrate-complex (LCC), aliphatic chain modification, polymer blend, biodegradability, compatibility

## Abstract

The purpose of this study is to modify lignin for better blending with general purpose synthetic polymers. The possible advantages by using this modification would be cost reduction, better physical properties, and biodegradability. In this study, butyrolactone-modified lignin (BLL) and tetrahydrofuran-modified lignin (THFL) were used for aliphatic chain modification of lignin using an acid-catalyzed esterification method in order to mimic the relation of lignin-carbohydrate-complex (LCC) and cellulose. The results of several analyses indicated that lignin was well modified. It was confirmed that the lignin was modified as expected and the reaction sites of the modification, as well as the reaction behaviors, were varied by the reagent types. The result of X-ray diffraction analysis (XRD) analysis indicated that modified lignin/polymer blends increased the crystallinity due to their good compatibility. It can be confirmed that the type of alkyl chain and the miscibility gap between the alkyl chain-matrix affected the mechanical properties enormously in the fungi-degradable environment. From this study, a new method of lignin modification is proposed, and it is found that modified lignin retains the property of the substituted aliphatic chain well. This method could be a proper lignin modification method.

## 1. Introduction

After the “Bronze Age”, modern society has evolved into the “Plastic Age”. There are many types and properties of plastics, for multiple purposes. Almost every plastic is difficult to degrade, requiring a long time, and causing environmental harm. Thus, raw plastic material from natural resources, without any environment problems, is necessary. In modern times, new plastic research or inventions focus on raw materials which are biodegradable or originated from nature. Therefore, it is necessary to find another natural resource, which is not used as food, have enough amount for industrial applications, and rarely or never used before and lignin may be a good candidate.

Wood is composed of three components; cellulose, hemicellulose, and lignin mixtures, which is called the ‘lignin-carbohydrate-complex’ (LCC) at the micro-scale. The purpose of any kind of pulping process is to breakup this LCC and use only cellulose [1]. Cellulose, hemicellulose, and every kind of materials which is extracted from wood are used, but not lignin. Kraft lignin is extracted from solution because it cannot be treated the way it is. Specifically, kraft lignin is treated with solutions containing high concentrations of alkali ions and neutralized by a strong acid to create a powder phase mixture which is composed of mostly lignin. Generally, this powder is burnt to supply the energy requirement of the production process [2]. Different from the kraft lignin which most of the materials are incinerated, lignosulfonate, organosolv lignin, and other lignins are used as feed binders, adhesives, emulsifiers, etc. However, all of these lignins are hard to use as wide-use materials, like synthetic polymers, due to its unique molecular structure and properties [3].

**Figure 1:**
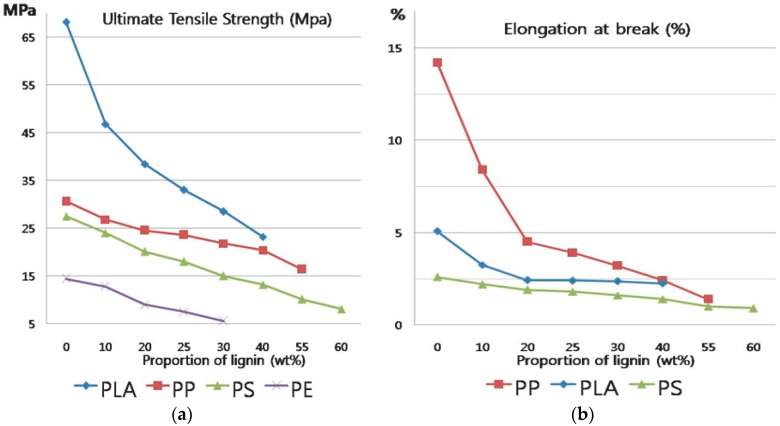
Tensile properties of lignin-various polymer blends. (**a**) Tensile Strength and (**b**) breaking strain [5,6,7,8].

Many researchers have reported that it is possible to produce thermoplastic materials which contain a large amount of lignin [4]. However, the elongation of such materials tends to decrease dramatically with increasing lignin content in fact, the physical properties of thermoplastic blends are significantly degraded when more than 10%~25% of lignin is incorporated into synthetic polymers, as shown in Figure 1 [5,6,7,8]. Therefore, maximizing the amount of lignin in thermoplastic materials while minimizing the corresponding degradation of mechanical properties is a very important research topic. The development of materials with such properties could reduce the price of production and environmental pollution.

Chemical modification of lignin is a major issue in the area of lignin research. Chemical modification of lignin is performed to replace the carboxyl group and hydroxyl group of the lignin by alkylation, acylation, and hydroxyalkylation [9]. A new method is proposed in this study where the lignin functional group is replaced by the proper molecular weight of an aliphatic chain. Replacing a lignin functional group is similar to another lignin modification method like alkylation and/or acylation, but the limitation of these methods is that those with molecular weight which are lower than one hundred Dalton can be substituted. The modification method used in this study faced difficulty in dramatically increasing compatibility between matrixes.

## 2. Experimental

### 2.1. Materials

Tetrahydrofuran and γ-butyrolactone were used in this study. These reagents and sulfuric acid which were used as catalysts were purchased from Junsei Co., Tokyo, Japan, GR grade. The Indulin AT^®^ (pine kraft lignin) used in this study was purchased from MeadWestVaco Co., North Charleston, SC, USA. Ingeo™. Polyethylene terephthalate (PET) (Mw = 44,650, Mn = 23,410, PDI = 1.91) with a low melting temperature—in order to blend with γ-butyrolactone modified lignin (BLL), polypropylene (PP) (Mw = 210,000, Mn = 49,400, PDI = 4.25)—in order to blend with tetrahydrofuran-modified lignin (THFL), were industrial grades and used as they were received. Sabouraud dextrose agar (model No: 210950) purchased from Becton and Dickinson Difco Co., Ltd. (Franklin Lakes, NJ, USA) (BD Difco) was used as a medium and *Aspergillus awamori* fungus (ATCC 6970) from the Korean Collection for Type Cultures (KCTC) (Daejeon, Korea) and were used for the degradation test.

### 2.2. Preparation of Modified Lignin

A modification procedure was started using fixed 5 mL reagent and 0.5 g~2.5 g of lignin, according to the 0.5 g variation, with charge ratio from 10:1 to 10:5. The reaction started with sulfuric acid as a catalyst and was put into a 1 wt % lignin/reagent mixture. BLL was reacted at 200 °C in the first half reaction time. After that, the temperature was increased to 250 °C and reacted for another half reaction time. The THFL was reacted first at 100 °C, and then reacted at 150 °C, like BLL. Agitation speed was 900 rpm for the entire process. The time variations were 10, 20, 30 min, 1 h, 1 h and 30 min, and 2 h, respectively, under evaporation in order to remove the byproduct and volatilized reagent. During the two hours of reaction, the viscosity of the mixture was increased rapidly in the last stage.

After following the determined time procedure, the reacted sample was washed with excess distilled water. The modified lignin sinking occurred due to its insolubility and higher density than the distilled water. A small amount of unreacted reagent, unreacted lignin, and catalyst were present as a suspended solid in the supernatant. After sinking, the supernatant was removed and unreacted material suspended to separate from modified lignin. The washed sample needed to be dried under vacuum conditions for use. In order to prevent a side reaction or post reaction due to the minimum quantity of remaining catalyst or reagent when the drying temperature is more than 100 °C, 12 h of vacuum drying must be proceeded at 80 °C with 30 in. hg, or 762 torr of pressure. To dry the sample completely, it was mixed in 3 h intervals.

**Figure 2:**
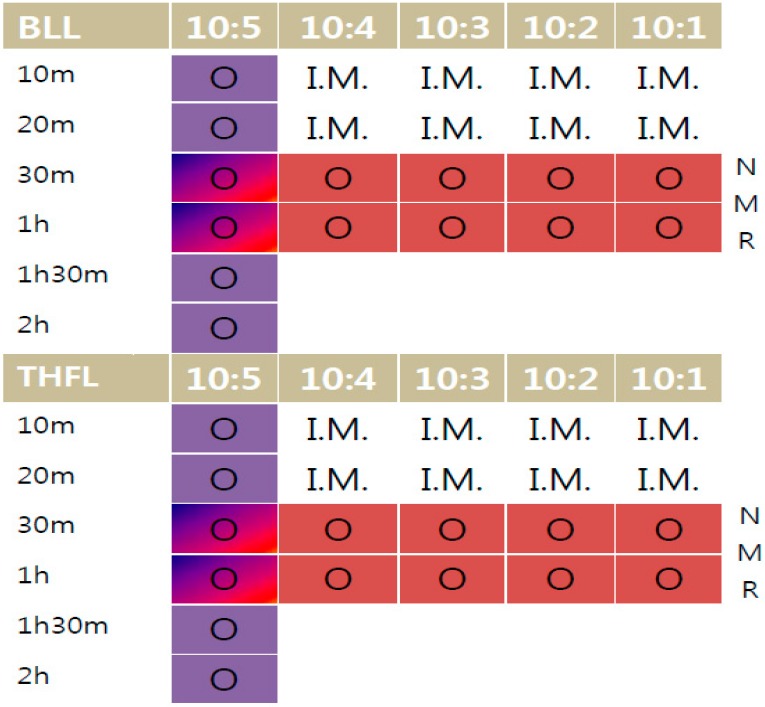
Prepared sample for analysis.

Figure 2 shows a modification time and reaction ratio variation. 10:X is the fixed reagent ratio versus lignin ratio, time is the whole reaction time of the sample. I.M. means ‘impossible mission’ due to too many unreacted materials. 31P-NMR analysis was performed on 30-min and one-hour reacted samples (red marked) and FT-IR analysis and solubility tests were performed on all samples.

### 2.3. Analyses of Modified Lignin Functional Group Using 31P-NMR and FT-IR

Thirty-minute modified lignin and one-hour modified lignin were monitored and analyzed by quantitative 31P-NMR using published procedures. NMR spectra were acquired using an AVANCE 600 (Bruker, Karlsruhe, Germany), 600 MHz spectrometer equipped with a quad probe dedicated to 1H, 15N, 19F, and 31P-NMR acquisition. To investigate the functional group change of modified lignin that have different reaction charge ratios and times, solid-state FT-IR spectra for the samples that have the fixed charge ratio, and time variations which were 10 min, 20 min, 30 min, one hour, and one hour and 30 min, respectively, were obtained with an FT-IR spectrophotometer (Nicolet Is 5, Thermo Scientific Inc., Waltham, MA, USA).

### 2.4. Analyses of Modified Lignin in Order to Investigate the Changes of Lignin Contents and Solubility

In order to investigate the solubility change of modified lignins that have different reaction charge ratios and times, lignin and modified lignin were dissolved in THF, which did not dissolve lignin. Each sample was dissolved in THF with a total concentration of 2%, (*w*/*v*, 100 mg/5 mL) for 5 min and, after that, they were centrifuged for 5 min at 1000 rpm. After removing the supernatant, surplus THF were volatilized in a vacuum chamber. Residues were weighed to quantify the insoluble fraction of modified lignin. The elemental contents of lignin and 2 h modified lignin were analyzed using a Thermo Electron Co., Waltham, MA, USA, Flash EA 1112-Elemental (C,N,S) Analyzer. Synthetic polymers and minerals, like sand or mineral salts, can be measured without error, although only with a few mg samples. However, natural materials like lignin or animal protein are difficult to measure without any error because they do not have regular empirical formulae. Therefore, lignin and modified lignin were weighed to more than 10 mg before measuring in order to reduce the error. Since the reacted reagent is composed of only carbon, oxygen, and hydrogen, and nitrogen only exists in lignin, the elemental analysis was conducted in order to compare the nitrogen content. 

### 2.5. Blending of Modified Lignin and Synthetic Polymers

Modified lignin was mixed with synthetic polymers in proportions of 0, 25.0, and 50.0 wt % of lignin content. The mixtures were melt blended using a table-type kneader (PBV-0.3, Irie Shokai Co., Tokyo, Japan) at 170 ± 2 °C for 20 min. Subsequently, the modified lignin/synthetic polymer composites were compression molded using a two-post manual hydraulic press (#2699, Carver Inc., Wabash, IN, USA) at 170 ± 1 °C and 6.89 MPa for 5 min. The molds were quenched after molding, and then polymer composite sheets were obtained.

### 2.6. Tensile Test

A universal testing machine (LRX Plus, LLOYD Instruments Inc., Bognor Regis, UK) was used to determine the tensile properties of the modified lignin/synthetic polymer composites and its degraded sample. Tensile tests were performed according to the guidelines of ASTM D638. The range of sample thickness was 1.2 to 1.8 mm, and the strain rate was 10 mm/min. The data are expressed with the averages of a minimum of 10 specimens.

### 2.7. X-ray Diffraction Analysis (XRD)

X-ray diffraction analysis was used to investigate the change of crystalline area by the modified lignin/matrix ratio (D8-discover with GADDS, Bruker, Germany). Basically, the determination is based on the relative intensity of specific peaks. The XRD method has been previously used to study the polymer structures and directly determine the crystallinity of polymers [10]. The determination of the degree of crystallinity thus involves the separation of the peaks scattered by the amorphous phase and the peak reflected by the crystalline phase. The degree of crystallinity can be computed using the equation:

(1) $$Xc=\frac{Ic}{Ic+IA}{\;}_{\;\times \;100\%}$$

where IC and IA are the scattered intensity for the crystalline and the amorphous phase, and XC is the degree of crystallinity of the blend. The degree of crystallinity was calculated by computerized integration values, using baseline integration software (Proteum 2, Bruker, Karlsruhe, Germany).

### 2.8. Degradation Test of Blends

**Figure 3:**
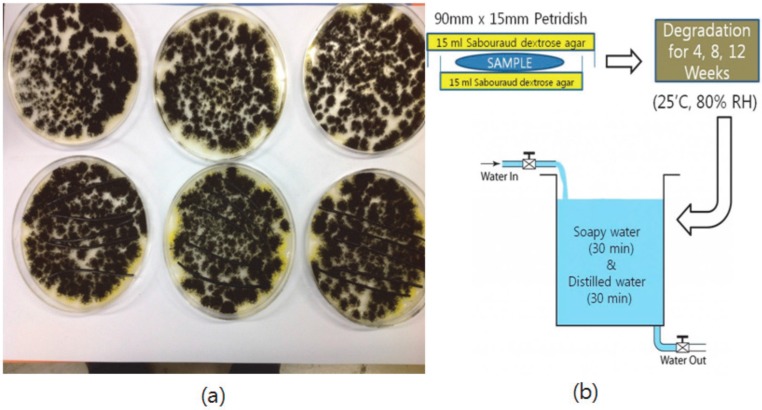
(**a**) Picture of fungi grown for 96 h at 25 °C and 80% RH and (**b**) scheme of the fungal degradation test.

The medium was prepared on the basis of the BD Difco’s manual [11] and was sterilized in an autoclave. Subsequently, both sides of a Petri dish (90 mm × 15 mm) were coated with 15 mL of medium, and the fungi were seeded. At a temperature of 25 °C and 80% RH (relative humidity), the fungi were grown for 96 h. Afterwards, the blend of 75% polymer and 25% modified lignin was placed on the middle of the medium coated surfaces (number of samples = 5). Fungi were allowed to degrade the sample for four weeks, eight weeks, and 12 weeks (Figure 3), and the samples were washed in soapy water for 30 min, then washed in distilled water for 30 min. The washed sample was stored in a desiccator for 24 h to remove the residual water. Finally, the change of the sample weight and mechanical properties were measured.

### 2.9. Scanning Electron Microscope (SEM) Image Analysis of Degraded Blends

The surface of the bio-degraded, modified lignin/synthetic blend was scanned by a SUPRA 55VP, Field-Emission Scanning Electron Microscope, Carl Zeiss, Germany. The degree of biodegradation was analyzed using 1000× images to show how modified lignin has an indirect effect on well-known, non-biodegradable polymers, like PET and PP.

## 3. Results and Discussion

### 3.1. 31P-NMR

Before quantification of modified lignin, determining a SKL functional group used in this study was necessary for this method since the lignin functional group is different according to the species of trees and/or extraction method. The position and amount of aliphatic OH (Al OH) and non-condensed aromatic OH (NC Ar OH) were determined to be 2.4 mmol/g and 2.2 mmol/g, respectively, from Hassan et al [12]. Furthermore, the main reaction sites of THFL, β-O-4 alpha hydroxyl group (β-O-4 OH) and COOH, were determined to be 2 mmol/g and 0.22 mmol/g, respectively, by several studies [13,14,15]. The homopolymer of polymerized reagent of butyrolactone is P4HB (poly-4-hydroxybutyrate) and polymerized tetrahydrofuran is poly tetramethylene glycol (PTMEG).

**Figure 4:**
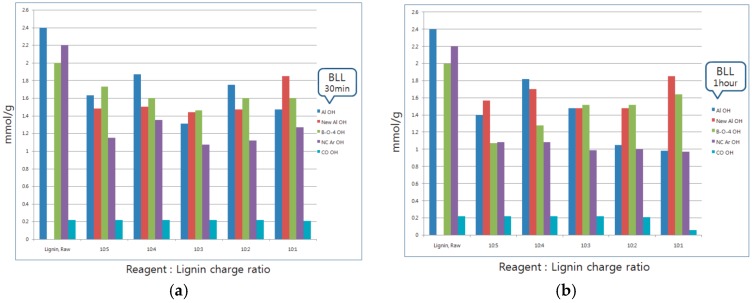
Changes of BLL functional groups according to the reaction charge ratio and reaction time.

BLL was mainly reacted with Al OH and NC Ar OH. A small amount of β-O-4 OH was reacted, although COOH did not react in BLL modification (Figure 4). Furthermore, NC Ar OH was reacted at the initial modification stage, and a decrease in Al OH was from chasing a NC Ar OH. The new aliphatic Al OH, in other words, increased the hydroxyl group at the end of the P4HB gradually. For BLL, the carboxyl group at the end of the P4HB chain that oligomerized the ring opening polymerization was reacted with the lignin hydroxyl group by trans-esterification, which is where another lignin hydroxyl group reacted with another P4HB oligomer. 

**Figure 5:**
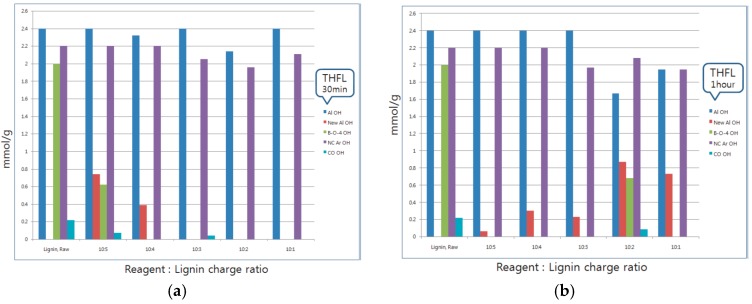
Changes of THFL functional groups according to the reaction charge ratio and reaction time.

**Figure 6:**
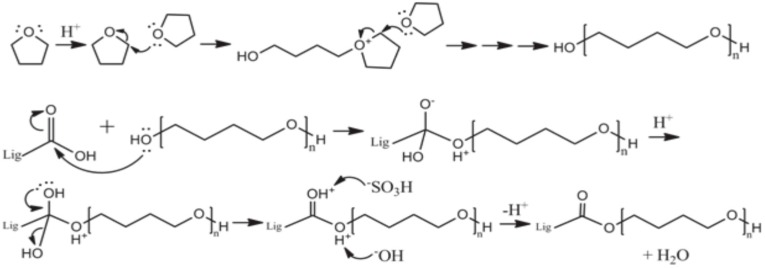
Reaction mechanism of modified lignin using an acid catalyst [22,23,24,25,26].

By contrast, in the case of THFL, almost all COOH and β-O-4 OH were decreased. THFL did not react to Al OH and NC Ar OH except when the reagent charge ratio was high. In Figure 5, new Al OH of THFL and OH at the end of the PTMEG were rarely present in THFL for 30 min. It is increased when the THFL sample reacted for one hour. This is because the hydroxyl group of oligomerized PTMEG was reacted with COOH and β-O-4 OH rapidly at the initial modification stage until, gradually, lignin-PTMEG copolymerization maximized. The specific reaction between THFL and β-O-4 OH is explained in the several references [16,17,18,19,20,21]. The COOH of lignin are reacted with OH of PTMEG by trans-esterification like in BLL. COOH is a well-known, highly-reactive functional group with electron delocalization, so the OH of PTMEG is reacted first to COOH. Then, as with BLL, the copolymer of lignin-PTMEG was gradually developed (Figure 6) [22,23,24,25,26].

### 3.2. FT-IR Spectra of Modified Lignin

**Figure 7:**
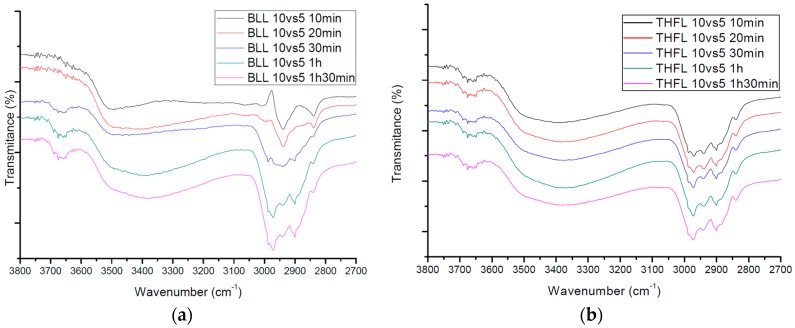
FT-IR spectra of (**a**) BLL and (**b**) THFL according to the reaction time.

The intramolecular OH bond peak is the distinguishing feature of modified lignin observed near 3650 cm^-1^. (Figure 7) This is rarely observed in lignin, unlike in materials like vanillin and/or cyclohexane. In lignin, it is the evidence of degraded intramolecular hydrogen bond by lignin-aliphatic chain copolymerization. 

In addition, an asymmetric stretch increases near 3000 cm^−1^~2900 cm^−1^ and features low molecular aromatic materials that look like lignin, according to the copolymerization of the lignin-aliphatic chain in progress. This peak is as shown as hydroxyalkylated lignin [27]. Carolina et al. studied the oxypropylation of lignin to use for polyols. Evidence of oxypropylation was checked by FT-IR analysis. Several features indicated the occurrence of propylene oxide grafting on lignin: an increase in the bands at 2971 cm^−1^~2870 cm^−1^ attributed to the asymmetric stretching of CH_3_, CH_2_, and CH in aromatic groups [27]. Making a connection between these two peaks, delocalized electron induced by carbonyl groups in the lignin-aliphatic chain increases the intramolecular hydrogen bond stretch, as opposed to the asymmetric stretch increasing the electron cloud in the copolymer aromatic ring captivated by electron delocalization.

### 3.3. Quantification of the Insoluble Fraction in THF

In order to quantify the modification of lignin, the insoluble fraction of modified lignin was quantified using the lignin insoluble solvent. The soluble property of lignin is insoluble in THF, the functional group of lignin had been substituted with a polymer chain that was used in the modification as shown in the various analyses, making the modified lignin soluble in the THF. The molecular structure of lignin was changed by modification, making the modified lignin more hydrophobic. The reason for lignin insolubility in THF, which is a hydrophobic organic solvent is due to the miscibility gap between lignin and THF by the excessive amount of hydroxyl groups of lignin. Table 1 shows the differences of insoluble fraction according to the reaction charge ratio and time. The same amount of unmodified lignin (2% (*v*/*w*)) has a 97%~98% insoluble fraction in the THF and this indicates the complete insolubility of lignin, considering the measuring error. However, BLL rarely shows an insoluble fraction, except for a 10:1 charge ratio. This phenomenon is due to the substituted chain that is used in the BLL modification is PHB, which has a solubility parameter (SP) value of 19.9, nearly closed SP value of THF (19.4) [28]. In the case of THFL, time variation rarely affected the differences of insoluble fraction while charge ratio was affected thoroughly. Furthermore, the insoluble fraction was increased in all charge ratios according to the time progression (30 min–1 h), because of the PTMEG chain that is used in the THFL-modified lignin had a more hydrophobic SP value than THF. It is said that PTMEG with 1000 Mn has a high hydrophobic SP value (17) [29].

As shown in the NMR analysis, degradation of the β-O-4 linkage occurred in the initial stage of the THFL modification process and substitution of PTMEG occurred at the same time. It is inferred that degraded lignin is alongside the substituted PTMEG chain. After that, the proportion of the PTMEG chain increased according to the time progression, and the insoluble fraction of THFL had increased linearly (Table 2). Furthermore, the lignin molecular structure was changed thoroughly from the structural point of view and the functional group of THFL is rarely different from lignin because THFL did not decrease the hydroxyl group of lignin compared with BLL, as supported by the FT-IR results. From these results, lignin was modified with the well-used modification method which was proposed in this study, and this can be inferred that BLL and THFL were modified into a similar structure, while the modification sites were different.

### 3.4. Elemental Analysis of 2 h-Reacted Lignin

In this section, the proportion of lignin in a modified lignin was analyzed by the proportion of nitrogen originated from lignin (Table 3). Considering the empirical formula of raw materials, the empirical formula of lignin is C_9_H_6.95_O_1.05_N_0.1_S_0.06_(OCH)_0.83_ However, the repeat unit of modification reagent is a BLL-C_4_H_6_O_2_, THFL–C_4_H_8_O. It can be easy to think that a low proportion of lignin means high aliphatic chain content, but it has low carbon and high oxygen content.

**Figure 8:**
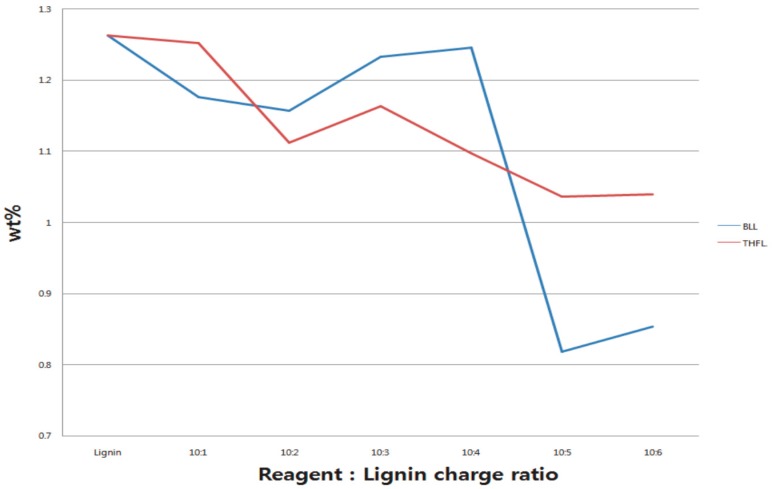
Decrease of nitrogen contents of modified lignin according to the reaction charge ratio.

For modified lignin for a ratio of 10:2~10:4, the aliphatic chain plays the role as the chain extender between lignins, as inferred from elemental analysis data. However, as shown in Figure 8, the proportion of aliphatic chains did not show linear changes and is gradually decreased as the charge ratio of lignin increased. The reason for this behavior can be explained by several studies of wood liquefaction. [30,31,32] The results of wood liquefaction showed that polycondensation took place when the charge ratios were 2 and 4, while polycondensation was not observed under the larger charge ratios, such as 6 and 10. The residue of liquefied wood is insoluble in DMF, which had resulted in the polycondensation [33]. From these results, degradation of lignin mainly occurred in the initial stage of modification, polycondensation occurred according to the time progress, and molecular weight increased rapidly at the end of the modification process. Furthermore, polycondensation occurrence needs a proper reaction condition, such as charge ratio, reaction time, catalyst ratio and type of raw materials. From these results, a 2 h reacted sample with a ratio of 10:6 is appropriate for the basic purposes of this study because of the low unreacted reagent and high lignin modification yield. A fixed condition of reacted lignin was used in the modified lignin/polymer blend section.

### 3.5. XRD Analysis of Modified Lignin/Polymer Blends

The blend of BLL/PET showed an increase in crystallinity more than PET only, and the reduction of crystallinity in the THFL/PP blend was rarely decreased. The THFL/PP blend showed a gradual decrease in crystallinity, whereas the crystallinity of the BLL/PET blend increased gradually because THFL acted as a filler (Table 4). However, the increase in crystallinity of PET due to the BLL addition is strong evidence for the good compatibility between BLL and PET.

**Figure 9:**
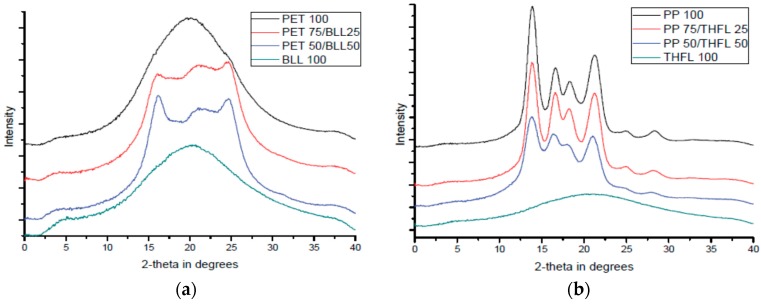
Changes of the crystal structure according to the (**a**) BLL and (**b**) THFL contents.

Furthermore, the crystal structure change of PET by the addition of BLL, as shown in Figure 9, means that molecular chain of BLL and PET are enough to compatibly entangle with each other. Additionally, it indicates the high compatibility of the PET/BLL blend along with increasing of the degree of crystallinity and peak shift of Tg in the DSC analysis. However, the PP/THFL blend did not show a dramatic change like the PET/BLL blend.

### 3.6. Tensile Test of 2 h-Modified Lignin and Polymer Blends

All of the mechanical properties of the PP/THFL blend were enhanced compared to lignin or the acetylated lignin/ polymer blend, which was confirmed by comparing their mechanical properties in this study, as well as other studies [35,36]. When modified lignin is incorporated into a PP matrix, the overall physical properties of the resulting composite are improved, such as when clay or talc is integrated into a general PP composite. Table 5 shows that the tensile properties of the PP/THFL blended matrix slightly decreased with the THFL content compared to the PP/lignin blend [5,6,7]. There was no significant change in the tensile strength, although the tensile strength of the matrix containing 50% THFL was slightly lower than that of the matrix containing 25% THFL. On the other hand, the tensile strength of the PET/BLL blend decreased as BLL content increased because P4HB is highly elastic and, thus, had a low modulus of 70 MPa [37]. In the case of the PET/BLL blend, the stress-strain seemed to follow the rule of mixture. Young’s modulus values decreased with the increase of BLL content from 1145 MPa to 126 MPa. However, elongation increased significantly with the increased concentration of BLL while the tensile strength decreased with the decreased concentration of PET. This result was attributed to the fact that P4HB exhibits 1000% elongation [38]. Since the mechanical properties of the polymer mixture follow the “rule of mixture”, the mechanical properties of the PET/BLL blend seemed to exhibit a mechanical performance proportional to the BLL content. These results indicate that the material used to modify lignin retains its material properties and affects the mechanical properties of the final blend due to the decreased number of OH bonds in lignin. Furthermore, the aliphatic chains that replaced the OH groups of lignin were confirmed to reduce the brittleness of lignin.

### 3.7. Degradation Behavior of PP/THFL and PET/BLL

Table 6 shows the results of mechanical test and weight loss after degradation. There was no significant weight loss in the 12-week experiment [39]. However, there was an obvious decrease of strain in all of the samples. In the case of the PP/THFL blends, a small change in tensile strength, decrease in strain, and increase in modulus was observed. It seems that a small change has occurred in the PP/THFL blend material during the degradation test because the THFL and PP were separated in the blend.

However, in the case of the PET/BLL blend, the tensile strength and strain decreased dramatically and the modulus increased more than double than that before degradation. This is because the chain degradation makes materials ‘brittle’, as shown in the bulk erosion process or photo-degradation of polymers [40,41]. The PET/BLL blend had a similar weight loss as it was shown in the PP/THFL blend. However, the PET/BLL blend showed good compatibility, which means that a chain of P4HB and PET is in a single phase lamella structure. After active biodegradation occurred, the lamella structure of the blend was weakened [42].

**Figure 10:**
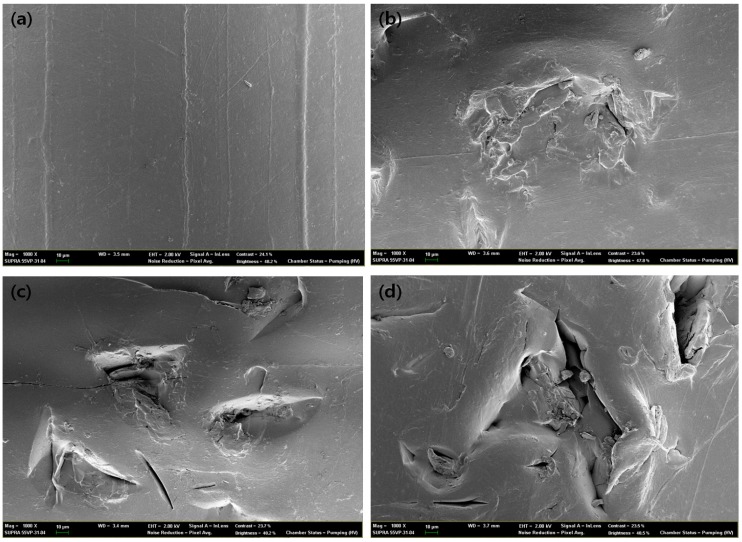
SEM pictures at the magnification of 1000× of PP/THFL 75/25 blend after degradation, (**a**) zero weeks; (**b**) four weeks; (**c**) eight weeks; and (**d**) 12 weeks.

**Figure 11:**
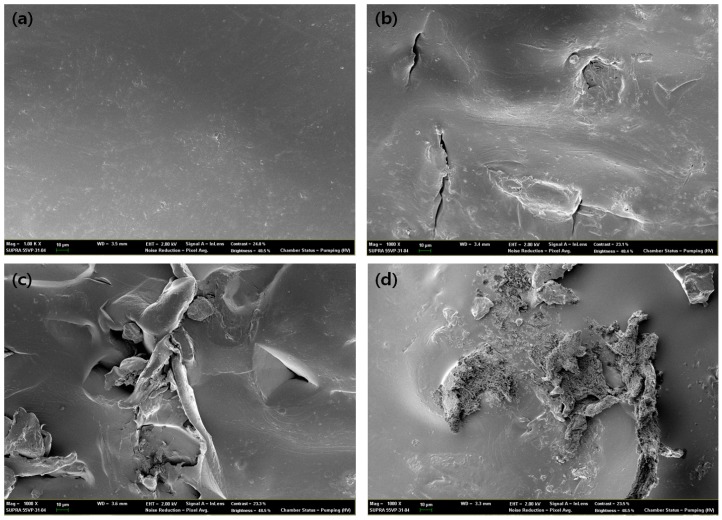
SEM pictures at the magnification of 1000× of PET/BLL 75/25 blend after degradation, (**a**) zero weeks; (**b**) four weeks; (**c**) eight weeks; and (**d**) 12 weeks.

Furthermore, Figure 10 and Figure 11 show 1000× degraded SEM images of modified lignin/PP and PET, which are well known non-degradable plastics. It can be shown that degradation occurred and increased as the time progresses, while the zero-week sample had shown smooth surfaces. If antioxidant was not added, lignin and substituted aliphatic chain degradation occurred with oxidative degradation not only of itself but also of the matrix polymer, as shown in a study of degradation behavior of lignin-blended PE and PP in a fungal degradation environment [43]. In the case of 12-week samples of the PET/BLL blend, it shows that the after-degraded product can be observed on the surface of a sample, while the PP/THFL blend degraded sample only shows burrowed defects into the surface. This means more active degradation occurred in the PET/BLL blend and it can be also confirmed by more decreased mechanical properties of the PET/BLL blend because P4HB is a well-known degradable polymer which can be used as a scaffold [37].

## 4. Summary and Conclusions

The purpose of this study was to propose an easy and inexpensive method of the modification of lignin. Various analyses were performed using different types of reagents to modify lignin. The reagents were used to mimic the LCC structure. The reaction site of modification was different with respect to the reagent, according to the results of 31P-NMR analysis. BLL was mainly reacted with Al OH and NC Ar OH. A small amount of β-O-4 OH was reacted. THFL did not react with Al OH and NC Ar OH, but reacted with COOH and β-O-4 OH rapidly at the initial modification stage. This is because of the low polarity of THF. β-O-4 linkage of lignin was degraded rapidly at the initial stage of modification, PTMEG substitution occurred at the same time. Polycondensation occurred in the last stage of modification. The yield of modification was higher when the charge ratio of lignin and the reaction time were higher.

XRD results showed the degree of crystallinity of the blend and interactions were different due to the types of the modified lignins and polymers. Tensile results indicate that the material used to modify lignin retains its material properties and affects the mechanical properties of the final blends, probably due to the decreased number of OH bonds in the lignin. In order to show the degradation behavior by addition of BLL and THFL, degradation test was performed for 12 weeks with four-week intervals. It was found that blends using BLL and THFL were degraded. BLL shows more active degradation. From this study, a new method of lignin modification is proposed, and it is found that modified lignin will retain the property of substituted aliphatic chains. This method could be a proper lignin modification method.

## Figures and Tables

**Table 1:** Insoluble fraction of modified lignin in THF according to the reaction charge ratio and reaction time variation.

Sample Label (wt %)	10:1	10:2	10:3	10:4	10:5
BLL 30 min	23.2	3.4	3.2	4	3.1
BLL 1 h	34.1	3.3	6.4	4.6	3.5
THFL 30 min	49.7	30.9	19.7	16.6	15.2
THFL 1 h	63.8	31.7	27.7	28.1	19.6

**Table 2:** Insoluble fraction of modified lignin in THF according to the reaction time variation.

Sample Label (wt %)	10 min	20 min	30 min	1 h	1 h 30 min
BLL 10:5	0.3	0.2	3.1	3.5	5
THFL 10:5	14.2	12	15.2	19.6	20.9

Raw lignin is completely insoluble in THF.

**Table 3:** Nitrogen components of modified lignin according to the reaction charge ratio.

Sample Label (wt %)	10:1	10:2	10:3	10:4	10:5	10:6
BLL	1.176	1.157	1.233	1.246	0.819	0.854
THFL	1.253	1.113	1.164	1.098	1.037	1.04

Raw lignin have a 1.2634 wt % of nitrogen.

**Table 4:** Degree of crystallinity of PP/THFL and PET/BLL blends [34].

Blend Type & Proportion (%)	100:0	75:25	50:50	0:100
PP/THFL	51.7	49.5	40	3.3
PET/BLL	30.1	35.4	33.4	10.9

**Table 5:** Tensile properties of polymer/modified lignin blends [34].

Blend Type & Proportion (%)	Ultimate Tensile Strength (MPa)	Young’s Modulus (MPa)	Elongation at Break (%)
PP 100	32.42 (±2.2)	711 (±143)	4.39 (±0.42)
PP75/THFL25	26.37 (±3.8)	712 (±192)	8.57 (±0.83)
PP50/THFL50	24.73 (±0.9)	959 (±154)	9.56 (±1.25)
PET 100	45.49 (±4.4)	1145 (±203)	5.33 (±0.02)
PET75/BLL25	23.23 (±2.7)	601 (±57)	447 (±72)
PET50/BLL50	7.26 (±1.3)	126 (±8)	503 (±87)

**Table 6:** Tensile properties and weight change of polymer/modified lignin—75/25 blends degraded by fungus [34].

Degradation Time (Week)	Ultimate Tensile Strength (MPa)	Elongation at Break (%)	Young’s Modulus (MPa)	Weight Loss (%)
PP/THFL 4W	19.7 (±1.6)	8.8 (±1.57)	634.8 (±239)	2
PP/THFL 8W	22.8 (±3.1)	5.6 (±0.77)	1031 (±173)	2
PP/THFL 12W	24.8 (±0.8)	5.5 (±0.21)	1264 (±175)	+1.3
PET/BLL 4W	12.1 (±2.1)	6.3 (±0.14)	1524 (±320)	1
PET/BLL 8W	9.8 (±0.6)	2 (±0.56)	1347 (±341)	0.5
PET/BLL 12W	9 (±0.4)	1.8 (±0.04)	1408 (±149)	+1.5
